# Targeted Deletion of the Metastasis-Associated Phosphatase *Ptp4a3* (PRL-3) Suppresses Murine Colon Cancer

**DOI:** 10.1371/journal.pone.0058300

**Published:** 2013-03-28

**Authors:** Mark W. Zimmerman, Gregg E. Homanics, John S. Lazo

**Affiliations:** 1 Department of Pharmacology and Chemical Biology, University of Pittsburgh School of Medicine, Pittsburgh, Pennsylvania, United States of America; 2 Department of Anesthesiology, University of Pittsburgh School of Medicine, Pittsburgh, Pennsylvania, United States of America; 3 Department of Pharmacology, University of Virginia School of Medicine, Charlottesville, Virginia, United States of America; University College London, United Kingdom

## Abstract

*Ptp4a3* (commonly known as PRL-3) is an enigmatic member of the *Ptp4a* family of prenylated protein tyrosine phosphatases that are highly expressed in many human cancers. Despite strong correlations with tumor metastasis and poor patient prognosis, there is very limited understanding of this gene family's role in malignancy. Therefore, we created a gene-targeted murine knockout model for *Ptp4a3*, the most widely studied *Ptp4a* family member. Mice deficient for *Ptp4a3* were grossly normal. Fewer homozygous-null males were observed at weaning, however, and they maintained a decreased body mass. Although *Ptp4a3* is normally associated with late-stage cancer and metastasis, we observed increased *Ptp4a3* expression in the colon of wildtype mice immediately following treatment with the carcinogen azoxymethane. To investigate the role of *Ptp4a3* in malignancy, we used the most commonly studied murine colitis-associated colon cancer model. Wildtype mice treated with azoxymethane and dextran sodium sulfate developed approximately 7–10 tumors per mouse in the distal colon. The resulting tumor tissue had 4-fold more *Ptp4a3* mRNA relative to normal colon epithelium and increased PTP4A3 protein. *Ptp4a3-*null mice developed 50% fewer colon tumors than wildtype mice after exposure to azoxymethane and dextran sodium sulfate. Tumors from the *Ptp4a3*-null mice had elevated levels of both IGF1Rβ and c-MYC compared to tumors replete with *Ptp4a3,* suggesting an enhanced cell signaling pathway engagement in the absence of the phosphatase. These results provide the first definitive evidence implicating *Ptp4a3* in colon tumorigenesis and highlight the potential value of the phosphatase as a therapeutic target for early stage malignant disease.

## Introduction

Protein tyrosine phosphatase-4a3 (*Ptp4a3*), along with *Ptp4a1* and *Ptp4a2*, comprise the 4a-family of protein tyrosine phosphatases. This modest gene family is commonly referred to as the phosphatases of regenerating liver (PRL), due to the discovery of *Ptp4a1* in the regenerating liver cells of mice following partial hepatectomy [1]. The significant homology (>75% amino acid identity) found among *Ptp4a* family members may denote similar enzymology but all three family members are conserved throughout mammalian species, suggesting non-redundant biological roles for each. The *in vivo* properties and functions of these enigmatic phosphatases remain remarkably poorly understood.

Interest in *Ptp4a3* can be attributed to its significant potential as a biomarker and as a therapeutic target for malignant cancers. Many human cancers express high PTP4A3 levels including tumors of the colon [2], breast [3], ovary [4], liver [5], stomach [6], and stroma [7], and elevated PTP4A3 expression often correlates with increased tumor invasiveness and poor prognosis [8]. Additionally, ectopic PTP4A3 overexpression enhances tumor cell migration and invasion *in vitro* [9]. While definitive evidence is lacking, *Ptp4a3* has been proposed to modulate multiple signaling pathways involving SRC [10], Rho GTPases [9], and PI3K-Akt [11] in various forms of cancer. The complexity of the pathway alterations seen when *Ptp4a3* is overexpressed may also reflect its ability to act as a phosphatidylinositol 5-phosphatase [12]. No reports have conclusively demonstrated a role for *Ptp4a3* in the physiology of normal cells or tissues.

Azoxymethane (AOM) is a procarcinogen that when metabolized in the colon is mutagenic and drives tumorigenesis. AOM in combination with the inflammatory agent dextran sodium sulfate (DSS) produces a widely-used murine model that faithfully replicates colon malignancies driven by chronic inflammatory conditions such as ulcerative colitis [13]. Several signaling pathways are implicated in the pathogenesis of AOM-induced colon cancer including KRAS [14], β-Catenin [15], c-MYC [16], Insulin-like Growth Factor-1 Receptor β (IGF1Rβ) [17], and Transforming Growth Factor β (TGFβ) [18]. Interestingly, *Ptp4a3* has been identified as a direct regulatory target of TGFβ signaling in colon cancer [19].

In the current study, we used a gene-targeting approach to generate mice lacking *Ptp4a3* and interrogate the potential role of *Ptp4a3* in colon tumorigenesis. AOM exposure acutely increased *Ptp4a3* expression in the colon. *Ptp4a3*-null mice were resistant to colon tumorigenesis implicating this gene in the pathogenesis of malignant disease. Moreover, tumors derived from *Ptp4a3*-null mice overexpresssed the cancer associated IGF1Rβ and c-MYC suggesting involvement of these oncogenic signaling pathways.

## Results

### Creation of Ptp4a3 mutant mice

**Figure 1:**
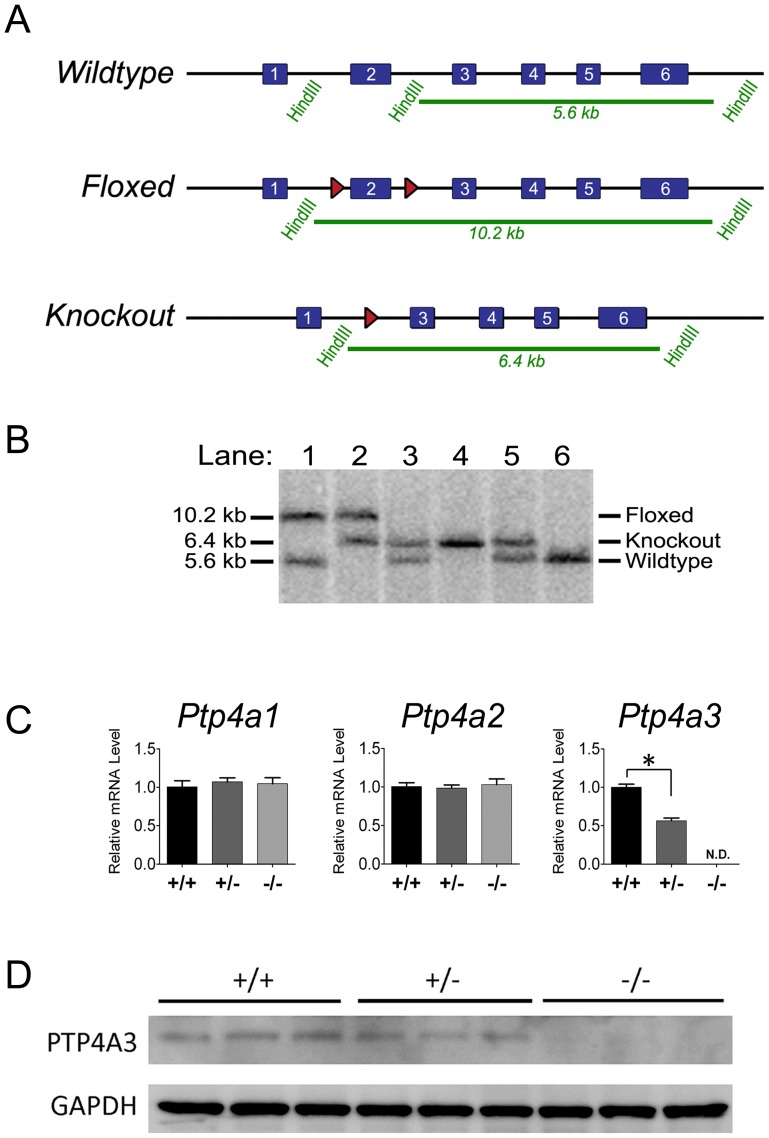
Gene-targeting the *Ptp4a3* locus. A) The *Ptp4a3* floxed allele was created by inserting loxP sites (red arrowheads) around exon 2. This mutation resulted in the disruption of a HindIII sequence that increases the size of the DNA fragment produced by restriction digest from 5.6 to 10.2 kb. Following expression of Cre recombinase, the sequence between the loxP sites is removed leaving a single loxP site and decreasing the size of the restriction fragment to 6.4 kb. This mutation results in deletion of the initiation codon causing a frameshift mutation producing a nonfunctional protein product. B) Southern blot analysis of genomic DNA was used to monitor these genetic changes and genotype mice containing the wildtype, floxed, or knockout alleles. Each lane is from an individual mouse. C) Quantitative RT-PCR analysis on total mRNA from fetal heart tissue revealed no change in *Ptp4a1* and *Ptp4a2* mRNA levels while *Ptp4a3* was reduced and not detectable in heterozygous and homozygous-null tissue, respectively. (* = p<0.05; n = 4/genotype). D) The PTP4A3 protein product was detectable by western blot in whole protein lysates from wildtype and heterozygous fetal heart tissue, but not in homozygous *Ptp4a3-*null samples.

We disrupted the *Ptp4a3* genomic locus using the Cre-lox system of site-specific recombination based on the position of two inserted loxP sites (Fig. 1A and further detail in Fig. S1). Mutant mice were backcrossed to the C57BL/6J strain for at least five generations. The knockout allele was created by crossing *Ptp4a3-*floxed mice with transgenic mice expressing CRE-recombinase driven from a general promoter (EIIA) to recombine the target site and create global knockout mice [20]. Insertion of the 3′ loxP site destroyed an endogenous HindIII site increasing the size of the genomic DNA fragment produced by restriction digest from 5.6 to 10.2 kb. Subsequent deletion of the target sequence reduced the restriction fragment size from 10.2 to 6.4 kb. Distinct band sizes corresponding to each allele are observed when analyzed by Southern blot hybridization with a ∼300 bp probe corresponding to exon 6 (Fig. 1B).

An analysis of >500 pups that were produced by heterozygous mating pairs indicated all potential genotypes were observed in the resulting offspring. While females were observed at the expected Mendelian ratios, we noted a significant decrease (p<0.05) in the number of *Ptp4a3*-null males at weaning relative to the predicted frequency (Table 1). Given this finding, it is possible that knockout males either possessed a survival disadvantage or that *Ptp4a3*-null germ cells had a preconception phenotype. *Ptp4a3*-null male mice exhibited an approximate 10% decrease in body mass (p<0.003) and 7% decrease in body mass index (p<0.004) compared to wildtype littermates at 6 wk of age (Fig. S2). This phenotype also appeared to be confined to male mice as female *Ptp4a3*-null mice did not exhibit a significant decrease in body mass or body mass index.

**Table 1:** Genotype distribution of Ptp4a3 mutant mice.

Offspring genotype at weaning						
Male	+/+	+/−	−/−	n	*X* ^2^	p
Observed	75	117	48	240	6.23	<0.05
*Expected*	*60*	*120*	*60*			

Female	+/+	+/−	−/−	n	*X* ^2^	p
Observed	70	127	79	276	2.34	N.S.
*Expected*	*69*	*138*	*69*			

There was a observed decrease in number of male *Ptp4a3*-null mice observed at weaning relative to the expected amount predicted by Mendelian genetics (p<0.05). Female mice of each genotype were born at the expected frequency (1:2:1).

Because fetal heart tissue had been previously suggested to have high levels of *Ptp4a3* expression [21], we next performed quantitative RT-PCR on total RNA samples from fetal heart tissue (E19.5) to determine the relative mRNA levels of *Ptp4a1*, *Ptp4a2,* and *Ptp4a3.* While *Ptp4a1* and *Ptp4a2* levels remained similar between genotypes, *Ptp4a3* mRNA was reduced in heterozygous tissue and not detectable in samples from *Ptp4a3*-null fetal heart tissue (Fig. 1C). Thus, compensation for *Ptp4a3* loss by upregulation of either family members *Ptp4a1* or *Ptp4a2* at the mRNA level was excluded. Protein lysates from fetal heart tissue were assayed by Western blot for the presence of the PTP4A3 protein product. While detectable in samples from wildtype embryos, PTP4A3 was lower in lysates from heterozygous embryos and homozygous-null tissue contained no detectable PTP4A3 (Fig. 1D). Aside from the aforementioned phenotypic observations, mice without functional *Ptp4a3* appeared grossly normal relative to wildtype littermates.

### Expression and knockout of Ptp4a3 in mouse tissues

Because the expression of endogenous PTP4A3 protein in normal tissues has not been well characterized, we examined tissue protein samples from wildtype and *Ptp4a3*-null mice. First, we assayed several tissue types for PTP4A3 protein expression by Western blot (Fig. S3). Fetal heart, fetal intestine, adult heart, skeletal muscle, pancreas, lung, spleen, brain, thymus, colon, and small intestine all expressed detectable levels of PTP4A3 in contrast to the liver and kidney, which had no detectable PTP4A3 protein. These results also confirm the efficacy of the global gene deletion approach, as no PTP4A3 protein was detectable in any of the lysates from *Ptp4a3*-null mice. Histological analysis was also performed on wildtype and *Ptp4a3*-null tissue samples (Fig. S4). Upon examining multiple tissue types, no overt abnormalities were observed and all samples appeared qualitatively normal.

### Ptp4a3 expression increases immediately following AOM exposure

**Figure 2:**
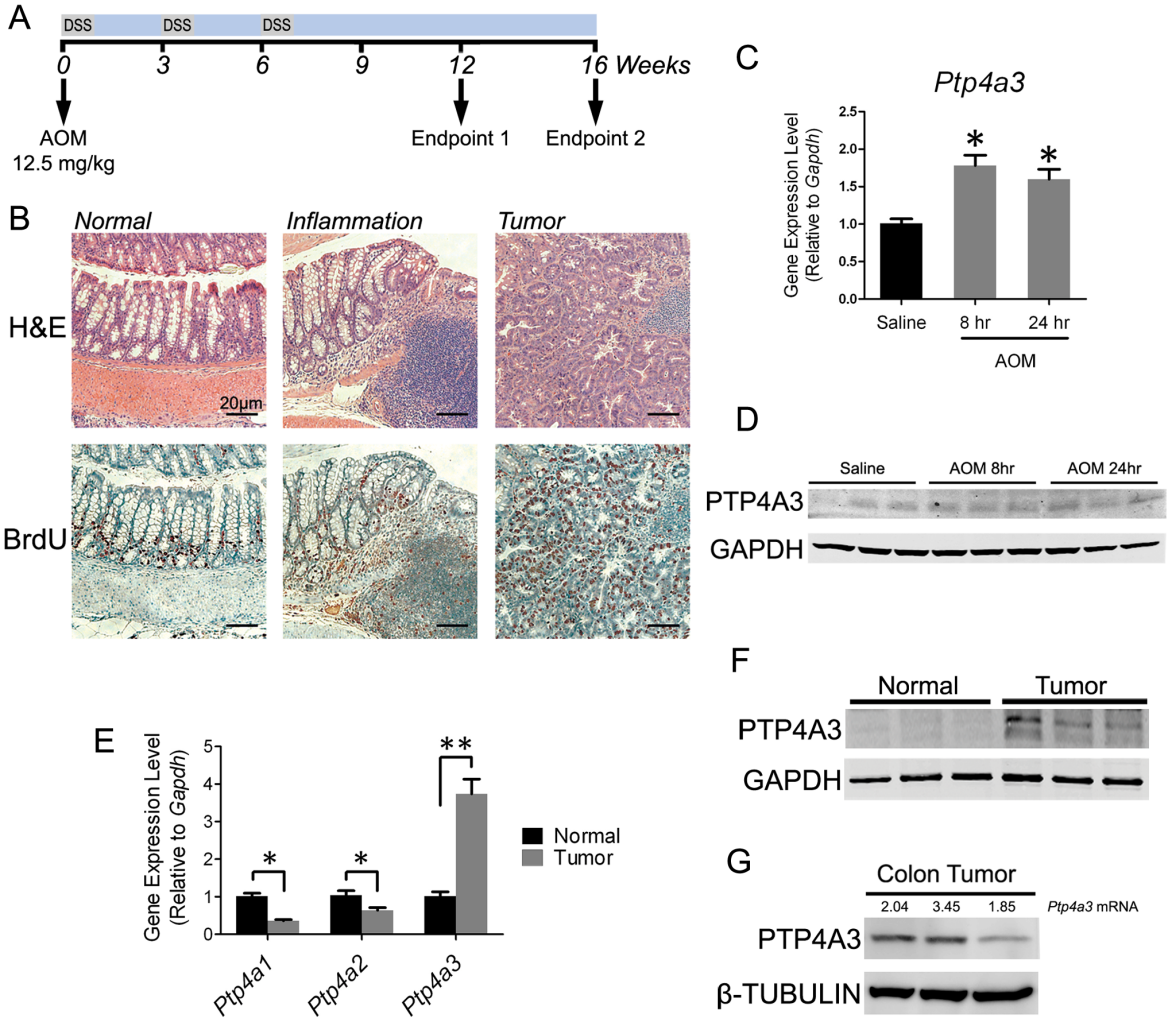
*Ptp4a3* is expressed in AOM-DSS derived colon tumors and normal tissue. A) The AOM-DSS treatment paradigm used in this study features a single dose of AOM followed by 3 treatment cycles of DSS in the drinking water. B) Histological representation of normal colon tissue relative to tumor tissue demonstrates the efficacy of the AOM-DSS model. Following one cycle of DSS treatment, crypt dysplasia and mononuclear cell infiltration were apparent. Tumor tissue was present at both the 12 and 16 wk endpoints. Before sacrifice, mice were treated with BrdU for 4 hrs and cell proliferation was visualized by staining with a BrdU antibody. C) Quantitative RT-PCR was used to assay *Ptp4a3* gene expression in normal colon epithelial tissue following treatment with either AOM or saline control. *Ptp4a3* was elevated 73% in colon tissue following when measured 8 hr after injection and 60% at 24 hr (* = p<0.001; n = 6/treatment group; bars  =  SEM). D) Western blot of colon lysates indicated an increase in PTP4A3 protein following AOM exposure. E) Quantitative RT-PCR indicated changes in gene expression levels of *Ptp4a* family members in normal colon and tumor tissue. *Ptp4a3* was elevated 3.7-fold (** = p<0.01), while *Ptp4a1* and *Ptp4a2* levels were significantly decreased (* = p<0.0001 and p<0.05, respectively) (n = 15, bars  =  SEM). F) Western blot analysis demonstrating higher PTP4A3 protein levels in colon tumors compared to normal tissue. G) Western blot combined with qRT-PCR analysis also demonstrated that when comparing *Ptp4a3* mRNA levels (listed above each lane) to PTP4A3 protein, high gene expression appeared to correspond to higher protein levels.

Since low levels of PTP4A3 were detectable in the normal colon epithelium, we assayed *Ptp4a3* gene expression immediately following treatment with the intestinal procarcinogen AOM (12.5 mg/kg) or saline control in wildtype C57BL/6J mice. Mice were sacrificed 8 and 24 hr after AOM injection and colon epithelial cells were collected for analysis. Total mRNA was isolated and quantitative RT-PCR was performed to assay for *Ptp4a3* gene expression levels. Interestingly, *Ptp4a3* was upregulated by 78% at 8 hr and 60% at 24 hr in AOM-treated normal epithelial relative to control (Fig. 2C). Protein lysates from AOM treated mice suggest that the PTP4A3 protein levels are also increased in these tissues (Fig. 2D). While the role of *Ptp4a3* in colon cancer is traditionally thought to involve late-stage tumors and metastasis, this finding suggests a potential role in early-stage disease and tumorigenesis.

### Knockout of Ptp4a3 suppresses intestinal tumor formation

**Figure 3:**
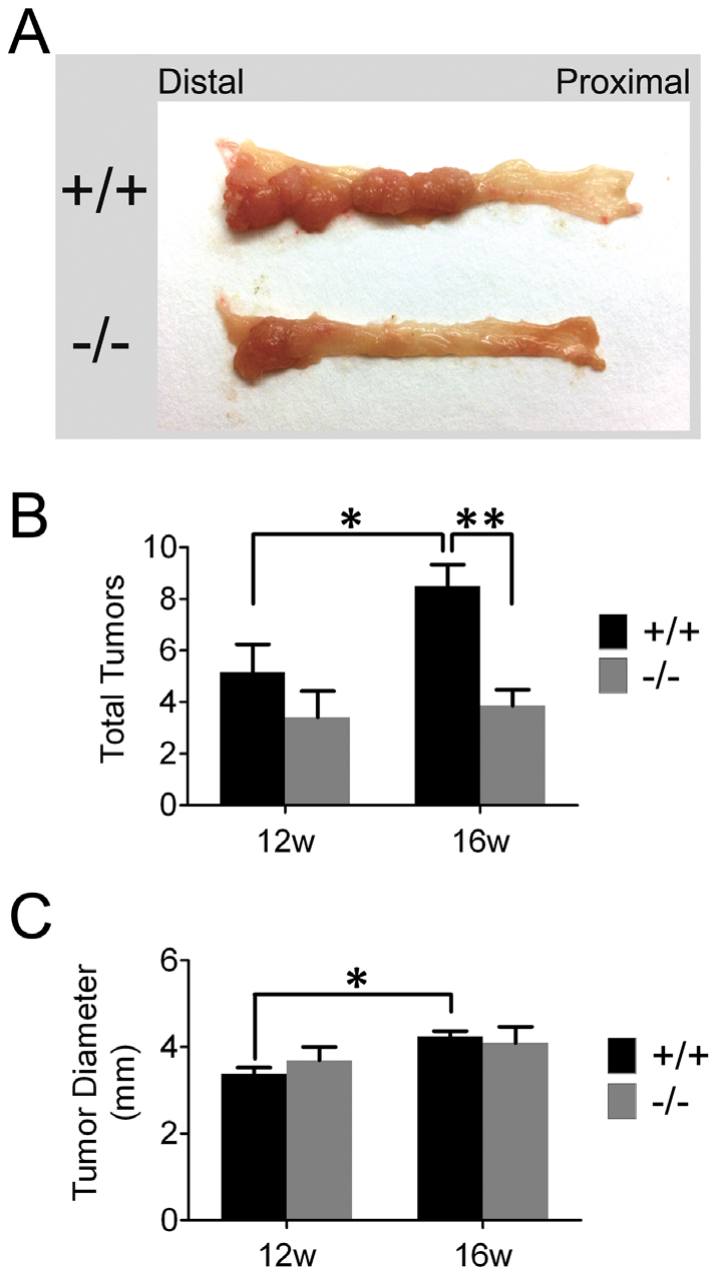
*Ptp4a3* knockout suppresses colon tumor formation. A) Image depicting the appearance of wildtype and *Ptp4a3*-null colon tissue following 16 wk treatment with AOM-DSS. B) The average number of tumors was recorded in mice by genotype after 12 and 16 wk of treatment. The average number of tumors significantly increased in wildtype mice between the 12 to 16 wk time points (* = p = 0.05). During this time the number of tumors observed in *Ptp4a3*-null mice was the same (p = 0.70). At 12 wks, wildtype mice (n = 6) did not have significantly more tumors per mouse than *Ptp4a3*-null (n = 5) mice (p = 0.27). At 16 wk, wildtype mice (n = 14) displayed significantly less tumors per mouse than *Ptp4a3*-null mice (n = 7) (** = p<0.005). C) Tumor size (measured by average tumor diameter for each mouse) was determined at each time point. Tumor diameter was observed to significantly increase in wildtype mice from 12 to 16 wks (* = p<0.001). No significant difference was observed in *Ptp4a3*-null mice from 12 to 16 wks, or between genotypes at either time point. Bars  =  SEM.

To explore a potential functional role for *Ptp4a3* in tumor formation, we subjected mice to a widely used AOM-DSS model of colitis-associated colon cancer. Wildtype and *Ptp4a3* – null mice were injected with a single dose of AOM (12.5 mg/kg) followed by three cycles of 2.5% DSS consumption (Fig. 2A). This model produces distinct histological changes relative to normal controls, including inflammation corresponding to DSS treatment, and subsequent dysplasia (Fig. 2B). Tumors were visually obvious in the distal colon of wildtype mice upon sacrifice at 12 or 16 wk after the initial AOM treatment and were (Fig. 2B and 3A). As indicated in Figure 3B, *Ptp4a3*-null mice exhibit a 54% decrease in tumor number (p<0.004) following 16 wk of treatment. Although on average fewer tumors were observed at 12 wk in *Ptp4a3*-null mice relative to wildtype, this was not statistically significant (p>0.2). Interestingly, the number of tumors (p = 0.05) as well as tumor diameter (P<0.001) was significantly increased from 12 to 16 wk, while *Ptp4a3*-null tumors did not significantly increase in number or size. The average diameter of tumors produced by this model was 3–4 mm (Fig. 3C).

### Ptp4a3 is elevated in AOM-derived colon tumors

Because high expression of PTP4A3 has been reported in human primary colon tumors [22], we examined *Ptp4a3* expression in the mouse model of colon cancer. First, total mRNA was extracted from tumor tissue from wildtype mice and quantitative RT-PCR was used to assay for the expression level of each *Ptp4a* family member (Fig. 2E). Relative to normal colon epithelium, *Ptp4a3* was elevated 3.7-fold on average (p<0.01). While considerable heterogeneity in *Ptp4a3* expression was observed, ranging from 1.4 to 6.7-fold upregulation, *Ptp4a3* mRNA levels in tumor tissue were consistently higher than normal tissue for every sample tested (n = 15). Interestingly, the gene expression levels of *Ptp4a1* and *Ptp4a2* were both downregulated 64% and 36% (p<0.0001 and p<0.05), respectively, in colon tumors relative to normal tissue. PTP4A3 protein levels in normal colon epithelial lysates were very low when assayed by Western blotting (Fig. 2F). In contrast, PTP4A3 was readily detectable, but variable, in colon tumor samples. As might be expected, there appeared to be some correlation between the PTP4A3 protein levels and the mRNA levels (Fig. 2G). The lack of functional antibodies for PTP4A1 and PTP4A2 precluded evaluating the tumor protein levels for these family members.

### Loss of Ptp4a3 increases IGF1Rβ and c-MYC expression in tumors

**Figure 4:**
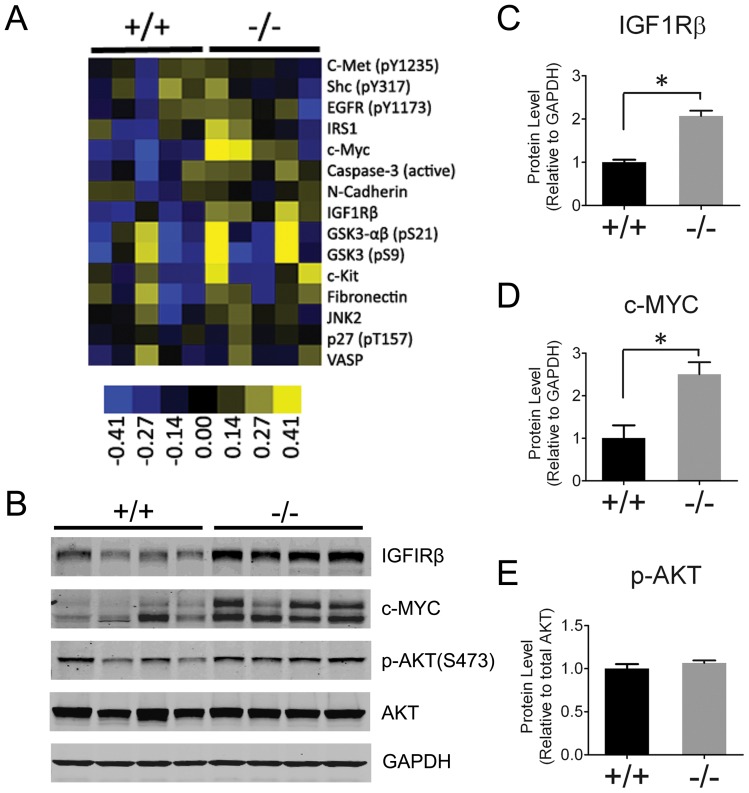
Higher IGF1Rβ and c-MYC levels in *Ptp4a3*-null tumors. A) Cluster sample of the heatmap generated from RPPA analysis that was performed using colon tumor lysates from AOM-DSS treated wildtype and *Ptp4a3*-null mice. Protein targets were either higher (yellow), lower (blue), or unchanged (black). B) Protein levels were confirmed by western blot analysis colon tumor lysates from wildtype and *Ptp4a3*-null mice. IGF1Rβ and c-MYC protein were chosen because of they appeared to be the most consistently upregulated proteins in *Ptp4a3*-null samples. C) When quantified, IGFIRβ protein levels were 2.1-fold higher in *Ptp4a3*-null tumors (p<0.001). D) c-MYC protein levels were 2.5-fold higher in *Ptp4a3*-null tumors (p<0.02). E) The levels of activated AKT, as indicated by phosphorylation of Ser473, were not significantly different by genotype relative to total AKT protein.

We next examined lysates obtained from both wildtype and *Ptp4a3*-null colon tumors. We first used reverse phase protein array to assay the levels of over 130 different protein products contained in wildtype and *Ptp4a3*-null tumors (Fig. S5). While protein levels in these tumors exhibited considerable heterogeneity, two known oncogenic signaling proteins, the receptor tyrosine kinase IFG1Rβ and the transcription factor c-MYC were expressed at higher levels in *Ptp4a3*-null tumors (Fig. 4A). Following quantification, IGF1Rβ protein was on average 2.1-fold higher (p<0.001) and c-MYC was about 2.5-fold higher (p<0.02) in *Ptp4a3*-null relative to wildtype colon tumors (Fig. 4B–C). In contrast, we did not observe a significant difference in AKT activation between genotypes (Fig. 4D). This result suggests that tumors can potentially compensate for *Ptp4a3* deficiency though altered signaling pathways.

## Discussion

A number of genes have been implicated in the development and progression of colorectal cancer. Previous gene expression profiling studies identified 144 genes that are up-regulated in metastatic colorectal liver samples [2]. The only gene, however, that was consistently elevated in all of the metastatic samples was *Ptp4a3*. Both gene amplification and enhanced transcriptional activity are likely causal for the high levels. Despite the apparent importance of *Ptp4a3* in tumor biology, our understanding of the functionality of *Ptp4a3* is severally limited due in part to the absence of informative animal models. The gene targeted mouse model for disruption of the *Ptp4a3* genomic locus that we have developed is useful not only for studying the phenotypic changes that occur with the global loss of the phosphatase, but also for future investigations on tissue and temporal specific gene deletion and collaborative interactions with other genes including the two other members of the *Ptp4a* family. Our model establishes that mice can survive in the absence of a functional *Ptp4a3* gene, although there were a slightly lower number of male mice produced, and they are able to live to maturity under normal conditions without any major health deficiencies. This is in contrast to what has been reported with mice lacking the highly homologous *Ptp4a2,* which is thought to be the most ubiquitously expressed family member under normal conditions. *Ptp4a2*-null mice exhibited defective placental development and both genders showed retarded growth at embryonic and adult stages [23]. *Ptp4a2* loss decreases the spongiotrophoblast and decidua layers of the placenta impairing nutrient transport and causing embryonic growth retardation. Moreover, loss of *Ptp4a2* results in AKT inactivation, which was not evident in our data regarding *Ptp4a3* loss. Collectively, the differences in the two gene deletion models are consistent with non-overlapping functions of these two close phosphatase family members.

Deletion of PTP4A3 protein was confirmed by Western blot analysis using a variety of tissue lysates from wildtype and *Ptp4a3*-null mice. Although initial studies had suggested *Ptp4a3* expression was restricted to heart, skeletal muscle, and pancreas [24], our data revealed a more ubiquitous expression pattern. While the highest levels of PTP4A3 were observed in fetal heart, the protein was also detectable at lower levels in adult heart, skeletal muscle, spleen, pancreas, brain, lung, thymus, colon, and small intestine; no PTP4A3 protein was detectable the liver or kidney.

Because human PTP4A3 is been implicated in the pathogenesis of human metastatic colorectal cancer, we investigated the effects of *Ptp4a3* deletion in a mouse model of colon cancer. Treatment with AOM/DSS is one of the most popular colon tumor mouse models and the C57BL/6J strain to which this model was backcrossed is particularly susceptible to colitis-associated cancer [13], [16], [17]. *Ptp4a3* is traditionally classified as a gene associated with metastasis and not known to be involved in the early stages of cancer progression. In the current study, we provide evidence that the *Ptp4a3* mRNA and PTP4A3 protein levels are increased soon after exposure to AOM, the initiating step in our colon cancer model. This is important evidence that PTP4A3 could be also involved in the preneoplastic stage of malignancy.

Mice subjected to the AOM/DSS model consistently developed tumors in the distal region of the colon. These primary tumors displayed consistently higher levels of *Ptp4a3* relative to normal colon epithelium – similar to what is seen in human colon cancer patients [22]. Notably, mice deficient for *Ptp4a3* had >50% reduction in tumor formation providing further evidence that *Ptp4a3* is a key mediator of colon cancer progression. Nevertheless, the complete absence of PTP4A3 phosphatase was insufficient to abolish colon tumorigenesis, suggesting that a subset of tumors may not require *Ptp4a3* as a driver of the disease. This is supported by the finding that not all tumors in this model had high (>2-fold upregulation) *Ptp4a3* expression levels.

The animal model we have developed presents a unique opportunity to address the role of *Ptp4a3* in tumor biology. AOM exposure causes DNA damage and genotoxic stress. A prominent response to DNA damage is induction of *p53*, which can induce *Ptp4a3* expression [25] and may provide an explanation for the induction of *Ptp4a3* in the colon of AOM treated mice. Additionally, *Ptp4a3* has been identified as a direct regulatory target of TGFβ signaling in colon cancer cells. The active TGFβ signal induces SMAD3/4 binding to the *Ptp4a3* genomic locus and thus inhibition of gene transcription [19]. Loss of TGFβ is a frequently observed phenomenon in human colon cancer [26], as well as the AOM mouse model of colon cancer [18]. It is likely that this event contributes to elevated *Ptp4a3* gene expression in cancer through SMAD3/4 inactivation. Interestingly, a mouse model deficient for *Smad3* has been reported to spontaneously develop colon cancer [27], and *Smad4* deficiency greatly exacerbates a mouse model of colon cancer [28]. The formation of neoplastic lesions in the *Ptp4a3*-null mice may be the consequence of the engagement of additional growth factor signaling pathways or oncogenes, such as IFG1Rβ and c-MYC. c-MYC is known to be increased in tumors after AOM/DSS treatment and both IFG1Rβ and c-MYC were markedly elevated in the *Ptp4a3*-null tumors relative to wildtype derived tumors.

While the *Ptp4a3* gene product initially was thought to be involved in tumor metastasis and late stage disease, our results provide evidence for possible roles at other stages of the disease including pre-neoplastic transformation and early carcinogenesis. The concept of therapeutically targeting *Ptp4a3* could, therefore, be attractive at multiple stages of cancer.

## Materials and Methods

### Creation of *Ptp4a3* mutant mice

The conditional gene-targeting vector was constructed using a phage-based *E. coli* recombination system [29]. Specific details regarding the vector construction and targeting strategy are described in Fig. S1. We specifically targeted exon 2 because it contained the transcriptional start site and was necessary for proper translation of the mRNA transcript. Following transfection of the construct into R1 embryonic stem cells [30], mice were created from correctly targeted clones using standard chimeric animal production techniques with C57BL/6J blastocysts. The novel strain was backcrossed to C57BL/6J (The Jackson Laboratory) for >5 generations and mice for experiments were produced using heterozygous breeding pairs. Genotyping was performed by Southern blot analysis with a radiolabeled probe corresponding to ∼300 bp of exon 6, which was external to the gene targeting vector. This study was carried out in accordance with the recommendations in the Guide for the Care and Use of Laboratory Animals of the National Institutes of Health. All relevant protocols were approved by the Institutional Animal Care and Use Committee of the University of Pittsburgh.

### AOM cancer model

Wildtype and *Ptp4a3*-null mice (male, 6–8 wks) were administered a single dose of AOM (12.5 mg/kg) (Sigma) in sterile saline by IP injection. DSS solution (2.5%) (MP Biomedicals) was given *ad libitum* for 7 d followed by 14 d of normal drinking water and this cycle was repeated a total of 3 times [13]. Experimental mice were euthanized at 12 or 16 wk following the initiation of treatment, at which point colon tissue was isolated, rinsed, and opened longitudinally for analysis. Tumor and normal tissue were either snap frozen in liquid nitrogen or submerged in 10% neutral buffered formalin. For each mouse, individual tumors were counted and measured with a digital caliper and average tumor count and diameter were determined for each genotype.

### Western blot analysis

Cells and tissues were lysed using RIPA buffer and quantified by Bradford assay. A total protein sample of 40 µg was separated using Novex SDS-PAGE reagents (Invitrogen) and transferred to nitrocellulose membranes. Membranes were blocked in Odyssey buffer (LiCor Biosciences) and incubated with primary antibodies overnight followed by secondary fluorescent antibodies according to the manufacturers' instructions. We used the following commercially available primary antibodies: PTP4A3 clone 318 (Santa Cruz Biotechnology), GAPDH, IGF1Rβ, c-MYC, p-AKT(S473), and AKT (Cell Signaling Technology).

### Quantitative RT-PCR

Total RNA was extracted from tissue using Trizol reagent as per the manufacturer's protocol (Invitrogen). A total of 500 ng of mRNA was converted to cDNA using the iScript first strand synthesis kit (Bio-Rad). The primers (Tab. S1) used for target amplification were diluted to a final concentration of 500 pM, and real-time monitoring of the PCR reaction was performed on a Biorad iQ5 thermocycler with 2X Sybr Green Mastermix (Bio-Rad). The following program was run for 40 cycles: 95°C for 0:30; 58°C for 1:00; and 72°C for 0:30.

### Reverse phase protein array

Protein lysates (100 ug each) from colon tumor samples (n = 5/genotype) were denatured and shipped frozen to MD Anderson Cancer Center (Houston, TX) for analysis. Briefly, lysates were two-fold-serial diluted for 5 dilutions and arrayed on nitrocellulose-coated slides, probed with antibodies, and visualized by diaminobenzidine colorimetric reaction. Relative protein levels for each sample were determined by interpolation of each dilution curves from the standard curve antibody slide. All the data points were normalized for protein loading and transformed to linear value. Linear values were transformed to Log2 value and then median-centered for hierarchical cluster analysis. The heatmap was generated in Cluster 3.0 as a hierarchical cluster using Pearson Correlation and a center metric (additional details are provided is Fig. S5).

### Statistics

Statistical analysis of offspring genotype was calculated by the Chi-squared test comparing observed and expected results. Data from cellular assays, western blot, and qRT-PCR quantifications was analyzed using the 2-tailed t-test. In both cases, significance was defined as p≤0.05.

## Supporting Information

Figure S1 (PDF)

Figure S2 (PDF)

Figure S3 (PDF)

Figure S4 (PDF)

Figure S5 (PDF)

Table S1 (PDF)

## References

[1] Diamond RH, Cressman DE, Laz TM, Abrams CS, Taub R (1994) PRL-1, a unique nuclear protein tyrosine phosphatase, affects cell growth. Mol Cell Biol 14: 3752–3762. 8196618 10.1128/mcb.14.6.3752-3762.1994 PMC358742

[2] Saha S, Bardelli A, Buckhaults P, Velculescu VE, Rago C, et al (2001) A phosphatase associated with metastasis of colorectal cancer. Science 294: 1343–1346. 11598267 10.1126/science.1065817

[3] Radke I, Götte M, Kersting C, Mattsson B, Kiesel L, et al (2006) Expression and prognostic impact of the protein tyrosine phosphatases PRL-1, PRL-2, and PRL-3 in breast cancer. Br J Cancer 95: 347–354. 16832410 10.1038/sj.bjc.6603261 PMC2360632

[4] Reich R, Hadar S, Davidson B (2011) Expression and clinical role of protein of regenerating liver (PRL) phosphatases in ovarian carcinoma. Int J Mol Sci 12: 1133–1145. 21541048 10.3390/ijms12021133 PMC3083695

[5] Zhao WB, Li Y, Liu X, Zhang LY, Wang X (2008) Evaluation of PRL-3 expression, and its correlation with angiogenesis and invasion in hepatocellular carcinoma. Int J Mol Med 22: 187–192. 18636172

[6] Ooki A, Yamashita K, Kikuchi S, Sakuramoto S, Katada N, et al.. (2011) Therapeutic potential of PRL-3 targeting and clinical significance of PRL-3 genomic amplification in gastric cancer. BMC Cancer 11. 10.1186/1471-2407-11-122 PMC3080833 21466710

[7] Nielsen TO, West RB, Linn SC, Alter O, Knowling MA, et al (2002) Molecular characterisation of soft tissue tumours: a gene expression study. Lancet 359: 1301–1307. 11965276 10.1016/S0140-6736(02)08270-3

[8] Guzińska-Ustymowicz K, Pryczynicz A (2011) PRL-3, an emerging marker of carcinogenesis, is strongly associated with poor prognosis. Anticancer Agents Med Chem 11: 99–108. 21291404 10.2174/187152011794941145

[9] Fiordalisi JJ, Keller PJ, Cox AD (2006) PRL tyrosine phosphatases regulate rho family GTPases to promote invasion and motility. Cancer Res 66: 3153–3161. 16540666 10.1158/0008-5472.CAN-05-3116

[10] Liang F, Liang J, Wang WQ, Sun JP, Udho E, et al (2007) PRL3 promotes cell invasion and proliferation by down-regulation of Csk leading to Src activation. J Biol Chem 282: 5413–5419. 17192274 10.1074/jbc.M608940200

[11] Wang H, Quah SY, Dong JM, Manser E, Tang JP, et al (2007) PRL-3 down-regulates PTEN expression and signals through PI3K to promote epithelial-mesenchymal transition. Cancer Res 67: 2922–2926. 17409395 10.1158/0008-5472.CAN-06-3598

[12] McParland V, Varsano G, Li X, Thornton J, Baby J, et al (2011) The metastasis-promoting phosphatase PRL-3 shows activity toward phosphoinositides. Biochemistry 50: 7579–7590. 21806020 10.1021/bi201095z

[13] Neufert C, Becker C, Neurath MF (2007) An inducible mouse model of colon carcinogenesis for the analysis of sporadic and inflammation-driven tumor progression. Nat Protoc 2: 1998–2004. 17703211 10.1038/nprot.2007.279

[14] O'Toole SM, Pegg AE, Swenberg JA (1993) Repair of O6-methylguanine and O4-methylthymidine in F344 rat liver following treatment with 1,2-dimethylhydrazine and O6-benzylguanine. Cancer Res 53: 3895–3898. 8358714

[15] Kohno H, Suzuki R, Sugie S, Tanaka T (2005) Beta-Catenin mutations in a mouse model of inflammation-related colon carcinogenesis induced by 1,2-dimethylhydrazine and dextran sodium sulfate. Cancer Sci 96: 69–76. 15723650 10.1111/j.1349-7006.2005.00020.x PMC11159258

[16] De Robertis M, Massi E, Poeta ML, Carotti S, Morini S, et al.. (2011) The AOM/DSS murine model for the study of colon carcinogenesis: From pathways to diagnosis and therapy studies. J Carcinog 10. 10.4103/1477-3163.78279 PMC3072657 21483655

[17] Kanneganti M, Mino-Kenudson M, Mizoguchi E (2011) Animal models of colitis-associated carcinogenesis. J Biomed Biotechnol 2011. 10.1155/2011/342637 PMC3025384 21274454

[18] Guda K, Claffey KP, Dong M, Nambiar PR, Rosenberg DW (2003) Defective processing of the transforming growth factor-beta1 in azoxymethane-induced mouse colon tumors. Mol Carcinog 37: 51–59. 12720300 10.1002/mc.10120

[19] Jiang Y, Liu XQ, Rajput A, Geng L, Ongchin M, et al (2011) Phosphatase PRL-3 is a direct regulatory target of TGFbeta in colon cancer metastasis. Cancer Res 71: 234–244. 21084277 10.1158/0008-5472.CAN-10-1487 PMC3064433

[20] Lakso M, Pichel JG, Gorman JR, Sauer B, Okamoto Y, et al (1996) Efficient in vivo manipulation of mouse genomic sequences at the zygote stage. Proc Natl Acad Sci U S A 93: 5860–5865. 8650183 10.1073/pnas.93.12.5860 PMC39152

[21] Guo K, Li J, Wang H, Osato M, Tang JP, et al (2006) PRL-3 initiates tumor angiogenesis by recruiting endothelial cells in vitro and in vivo. Cancer Res 66: 9625–9635. 17018620 10.1158/0008-5472.CAN-06-0726

[22] Molleví DG, Aytes A, Padullés L, Martínez-Iniesta M, Baixeras N, et al (2008) PRL-3 is essentially overexpressed in primary colorectal tumours and associates with tumour aggressiveness. Br J Cancer 99: 1718–1725. 19002188 10.1038/sj.bjc.6604747 PMC2584959

[23] Dong Y, Zhang L, Zhang S, Bai Y, Chen H, et al.. (2012) Phosphatase of regenerating liver 2 (PRL2) is essential for placenta development by downregulating PTEN (phosphatase and tensin homologue deleted on chromosome 10) and activating Akt. J Biol Chem (In press). 10.1074/jbc.M112.393462 PMC3442547 22791713

[24] Matter WF, Estridge T, Zhang C, Belagaje R, Stancato L, et al (2001) Role of PRL-3, a human muscle-specific tyrosine phosphatase, in angiotensin-II signaling. Biochem Biophys Res Commun 283: 1061–1068. 11355880 10.1006/bbrc.2001.4881

[25] Basak S, Jacobs SB, Krieg AJ, Pathak N, Zeng Q, et al (2008) The metastasis-associated gene Prl-3 is a p53 target involved in cell-cycle regulation. Mol Cell 30: 303–314. 18471976 10.1016/j.molcel.2008.04.002 PMC3951836

[26] Massagué J (2008) TGFβ in Cancer. Cell 134: 215–230. 18662538 10.1016/j.cell.2008.07.001 PMC3512574

[27] Zhu Y, Richardson JA, Parada LF, Graff JM (1998) Smad3 mutant mice develop metastatic colorectal cancer. Cell 94: 703–714. 9753318 10.1016/s0092-8674(00)81730-4

[28] Takaku K, Oshima M, Miyoshi H, Matsui M, Seldin MF, et al (1998) Intestinal tumorigenesis in compound mutant mice of both Dpc4 (Smad4) and Apc genes. Cell 92: 645–656. 9506519 10.1016/s0092-8674(00)81132-0

[29] Liu P, Jenkins NA, Copeland NG (2003) A highly efficient recombineering-based method for generating conditional knockout mutations. Genome Res 13: 476–484. 12618378 10.1101/gr.749203 PMC430283

[30] Nagy A, Rossant J, Nagy R, Abramow-Newerly W, Roder JC (1993) Derivation of completely cell culture-derived mice from early-passage embryonic stem cells. Proc Natl Acad Sci U S A 90: 8424–8428. 8378314 10.1073/pnas.90.18.8424 PMC47369

