# Supplementary material for: Targeted Deletion of the Metastasis-Associated Phosphatase Ptp4a3 (PRL-3) Suppresses Murine Colon Cancer
Source: PLoS One. 2013 Mar 28;8(3):e58300. doi: 10.1371/journal.pone.0058300 (PMC3610886; doi:10.1371/journal.pone.0058300)

**SUPPLEMENTAL DATA**

**Figure S1 –** *Strategy for targeting the Ptp4a3 gene locus*. A gene-targeting construct was created by retrieving a 9 kb fragment of murine strain 129X1 genomic DNA from a bacterial artificial chromosome (The Sanger Institute). The newly created targeting vector was transformed into *E. coli* and all cloning and recombination steps were performed in bacteria. First, a PGK driven neomycin resistance cassette (NEO) flanked by loxP sites (red arrows) was inserted between exons 1 and 2. Expression of CRE recombinase in these cells deleted the floxed region leaving behind a single loxP site. Next, a FRT flanked (orange arrows) PGK-NEO cassette adjacent to a single loxP site was inserted between exons 2 and 3. Following analysis by restriction digest and sequencing, this construct was electroporated into R1 embryonic stem cells (21). G418 and gancyclovir resistant ES cell clones were expanded and screened by Southern blot using a radio-labeled probe corresponding to ~300 bp of exon 6, which was external to the gene targeting vector. Correctly targeted clones were injected into C57BL/6J 3.5-day blastocysts and mice were created using standard chimeric animal production techniques. The NEO cassette was subsequently removed by crossing to a strain expressing FLPe recombinase from a general promoter (actin). The floxed allele was then crossed to a strain expressing CRE recombinase also from a general promoter (EIIA). The novel mutant strain was backcrossed to C57BL/6J (The Jackson Laboratory) for at least 5 generations and mice for experiments were produced using heterozygous breeding pairs.

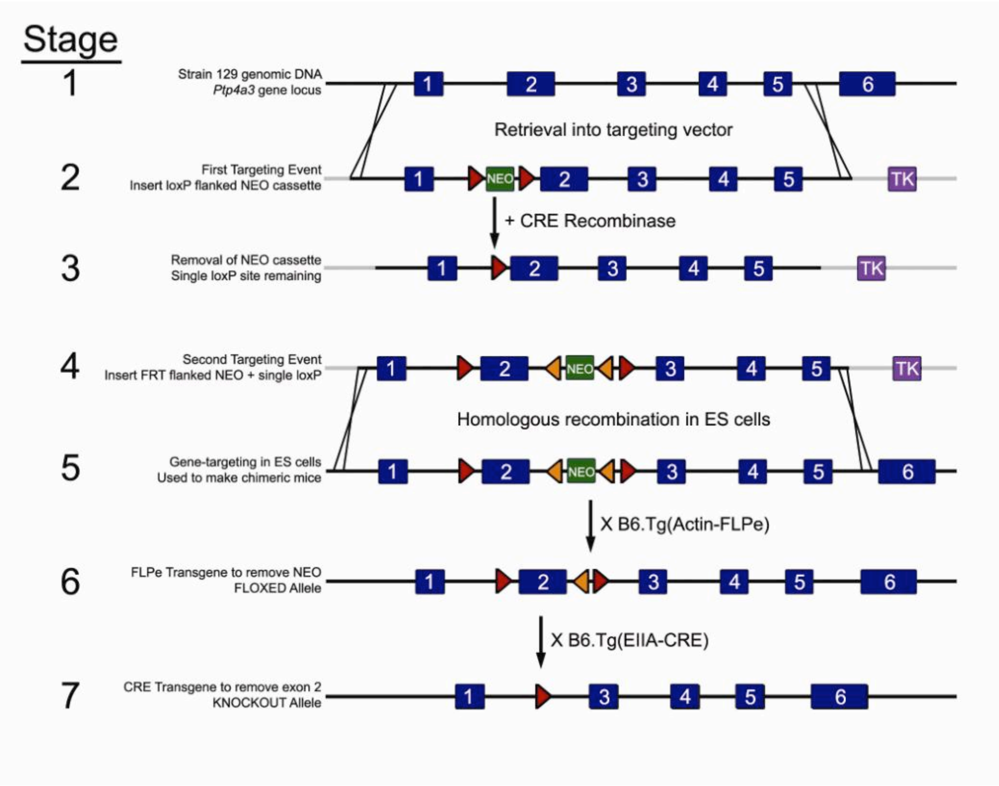

Supplement: Figure S1 — (PDF) [file pone.0058300.s001.pdf]
