# Supplementary material for: Targeted Deletion of the Metastasis-Associated Phosphatase Ptp4a3 (PRL-3) Suppresses Murine Colon Cancer
Source: PLoS One. 2013 Mar 28;8(3):e58300. doi: 10.1371/journal.pone.0058300 (PMC3610886; doi:10.1371/journal.pone.0058300)

Figure S2 - *Male Ptp4a3 knockout mice exhibited decreased weight and body mass index*. A) When compared to wildtype littermates (n=9/genotype), male *Ptp4a3*$^{-/-}$ mice exhibited an average of ~10% less body mass (p<0.005). B) Mice were also measured from the nose to the base of the tail and body mass index was determined (BMI=kg/m^2). Male *Ptp4a3*$^{-/-}$ littermates exhibited a ~7% decrease in BMI compared to wildtype (p<0.005). Neither of these phenotypes were significantly altered in female *Ptp4a3*$^{-/-}$ mice compared to wildtype.

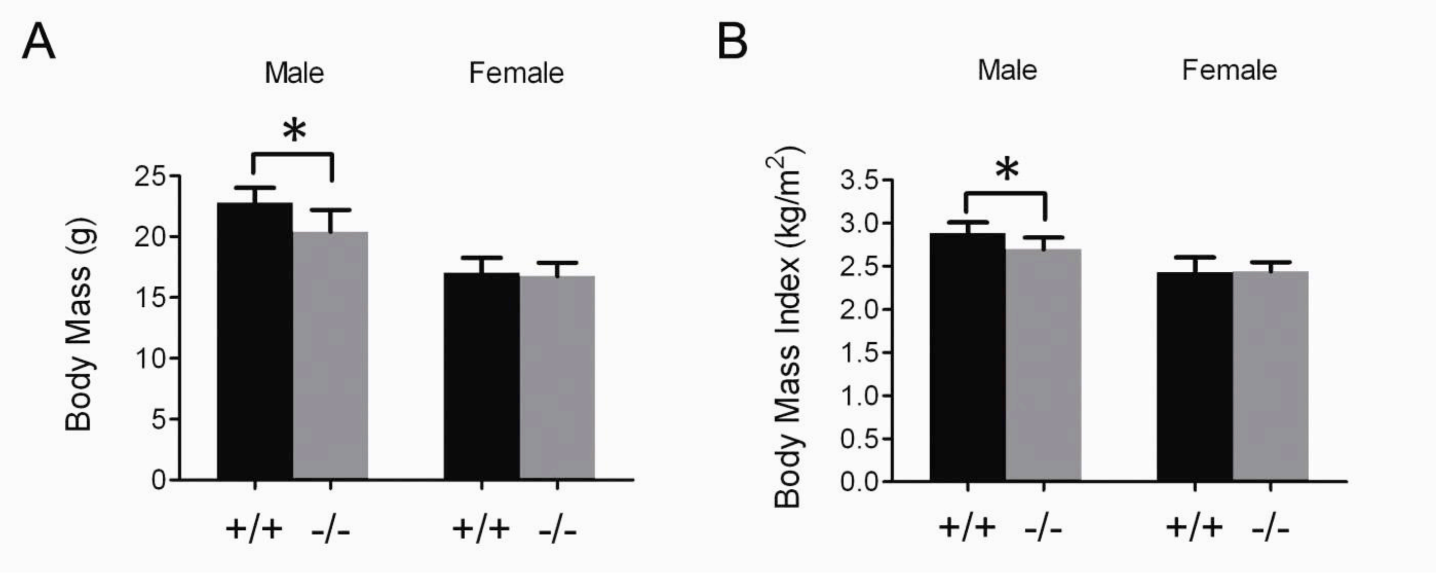

Supplement: Figure S2 — (PDF) [file pone.0058300.s002.pdf]
