# Supplementary material for: Targeted Deletion of the Metastasis-Associated Phosphatase Ptp4a3 (PRL-3) Suppresses Murine Colon Cancer
Source: PLoS One. 2013 Mar 28;8(3):e58300. doi: 10.1371/journal.pone.0058300 (PMC3610886; doi:10.1371/journal.pone.0058300)

Figure S3 - *Histological analysis of wildtype and Ptp4a3-null tissues*. Freshly isolated tissues were submerged in 10% neutral buffered formalin (Sigma) and fixed at room temperature overnight. Tissues were dehydrated through an ethanol gradient, embedded in paraffin, and sectioned onto glass slides. Sections stained with hematoxylin and eosin appeared qualitatively similar between genotypes (bar=5 μm).

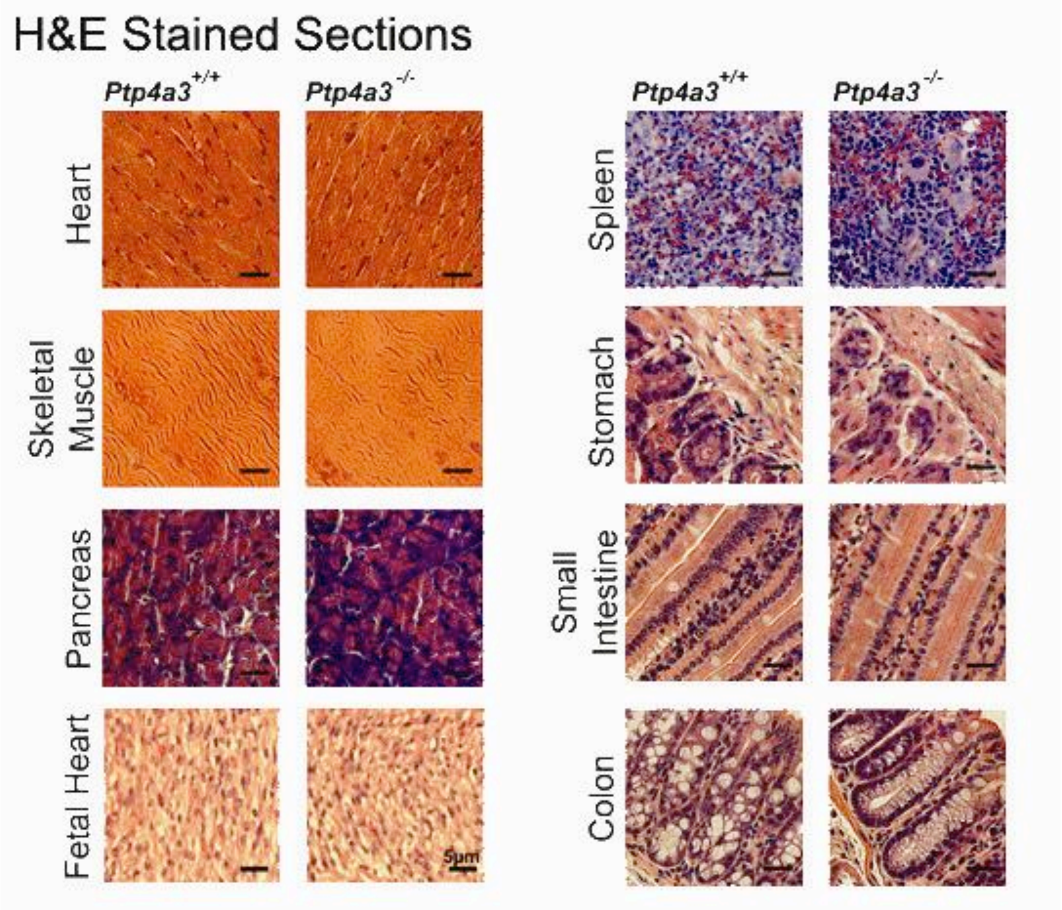

Supplement: Figure S3 — (PDF) [file pone.0058300.s003.pdf]
