# Supplementary material for: Targeted Deletion of the Metastasis-Associated Phosphatase Ptp4a3 (PRL-3) Suppresses Murine Colon Cancer
Source: PLoS One. 2013 Mar 28;8(3):e58300. doi: 10.1371/journal.pone.0058300 (PMC3610886; doi:10.1371/journal.pone.0058300)

Figure S4 - *Expression and knockout of PTP4A3 in mouse tissues*. Western blot analysis was performed on whole organ lysates from adult wildtype mice (n=3) with *Ptp4a3*$^{-/-}$ lysates used as a negative control (n=1). We then assayed for expression of PTP4A3 protein in various mouse tissues with GAPDH as a loading control. While the highest levels of PTP4A3 were observed in lysates from fetal heart tissue, the protein was also detectable at lower levels in samples from adult heart, skeletal muscle, spleen, pancreas, brain, lung, thymus, colon, and small intestine. The liver and kidney were the only tissue types analyzed that exhibited little or no expression of PTP4A3 protein.

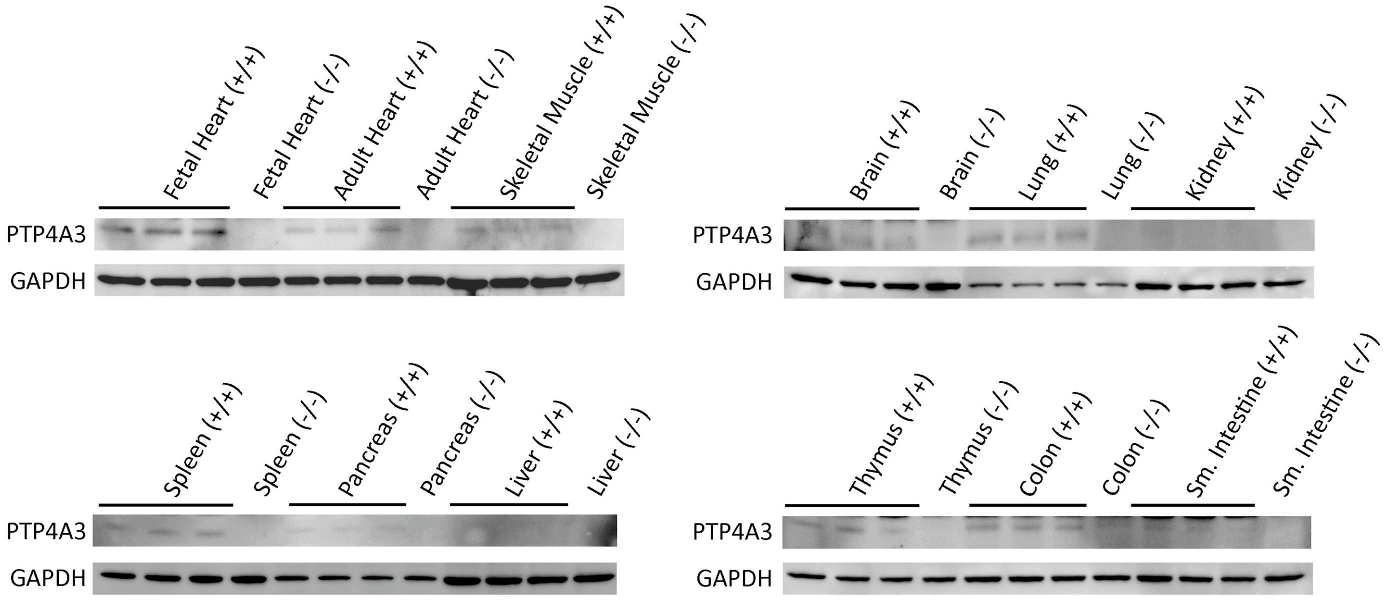

Supplement: Figure S4 — (PDF) [file pone.0058300.s004.pdf]
