# Supplementary material for: Targeted Deletion of the Metastasis-Associated Phosphatase Ptp4a3 (PRL-3) Suppresses Murine Colon Cancer
Source: PLoS One. 2013 Mar 28;8(3):e58300. doi: 10.1371/journal.pone.0058300 (PMC3610886; doi:10.1371/journal.pone.0058300)

Figure S5 – *Reverse phase protein array (RPPA) analysis*. Protein lysates (100 ug each) from colon tumor samples (n=5/genotype) were denatured and shipped frozen to MD Andersen Cancer Center (Houston, TX) for analysis. Briefly, lysates were two-fold-serial diluted for 5 dilutions and arrayed on nitrocellulose-coated slides, probed with antibodies, and visualized by DAB colorimetric reaction. Relative protein levels for each sample were determined by interpolation of each dilution curves from the standard curve antibody slide. All the data points were normalized for protein loading and transformed to linear value. Linear values were transformed to Log2 value and then median-centered for hierarchical cluster analysis. The heatmap was generated in Cluster 3.0 as a hierarchical cluster using Pearson Correlation and a center metric.

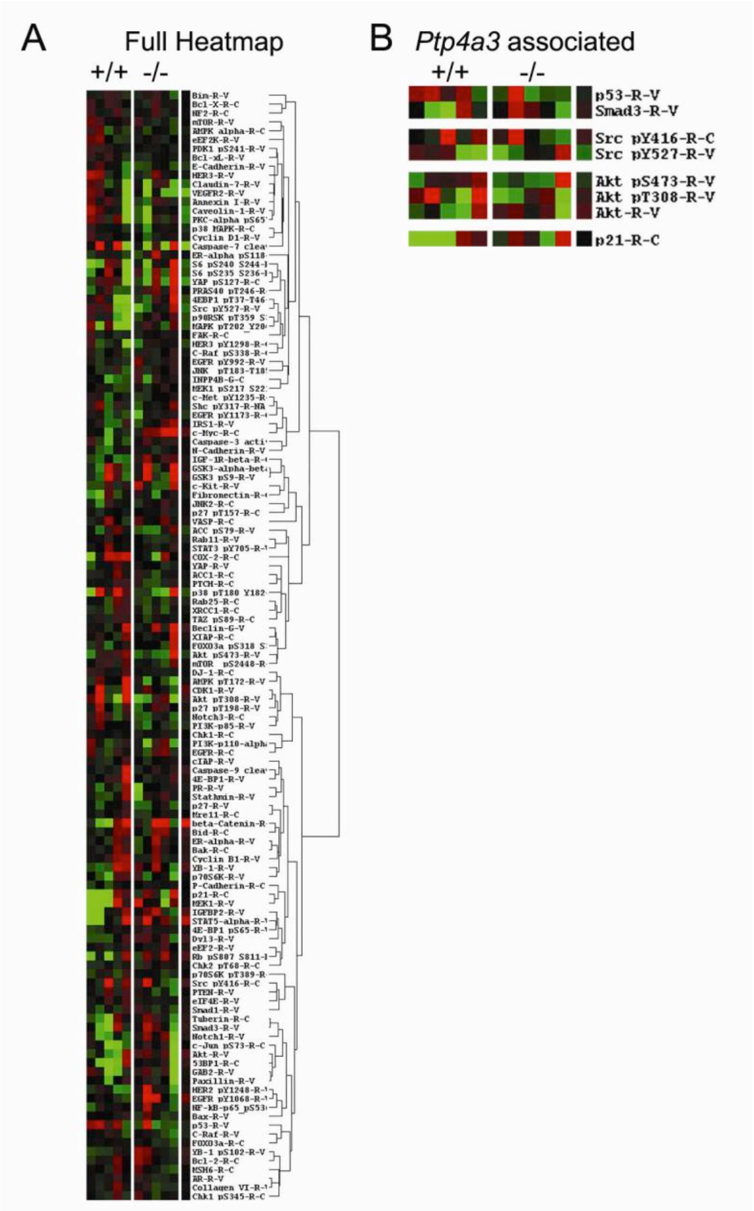

Supplement: Figure S5 — (PDF) [file pone.0058300.s005.pdf]
