# Supplementary material for: Targeted Deletion of the Metastasis-Associated Phosphatase Ptp4a3 (PRL-3) Suppresses Murine Colon Cancer
Source: PLoS One. 2013 Mar 28;8(3):e58300. doi: 10.1371/journal.pone.0058300 (PMC3610886; doi:10.1371/journal.pone.0058300)
Supplement: Table S1 — (PDF) [file pone.0058300.s006.pdf]

Table S1 - *Primers used for quantitative RT-PCR*. Listed are the forward and reverse primer sequences used to amplify each *Ptp4a* target gene as well as the *Gapdh* endogenous control.

**Target Gene**	**Forward Primer**	**Reverse Primer**
Ptp4a1	CAACCAATGCGACCTTAA	CAATGGCATCAGGCACCC
Ptp4a2	ATTTGCCATAATGAACCG	ACAGGAGCCCTTCCCAAT
Ptp4a3	CTTCCTCATCACCCACAACC	TACATGACGCAGCATCTGG
Gapdh	AACGACCCCTTCATTGAC	TCCACGACATACTCAGCAC
